# Telerehabilitation to Address the Rehabilitation Gap in Anterior Cruciate Ligament Care: Survey of Patients

**DOI:** 10.2196/19296

**Published:** 2020-09-18

**Authors:** Emma Dunphy, Elizabeth C Gardner

**Affiliations:** 1 eHealth Unit, Department of Primary Care and Population Health University College London Rowland Hill Street United Kingdom; 2 Department of Orthopaedics and Rehabilitation Yale University School of Medicine 47 College St New Haven, CT United States

**Keywords:** anterior cruciate ligament, telerehabilitation, rehabilitation, eHealth, knee, survey, telehealth, patient experience

## Abstract

**Background:**

Evidence shows that after anterior cruciate ligament (ACL) reconstruction, patients may have varied access to physical therapy. In particular, physical therapy input may end many months before patients reach full recovery. Telerehabilitation may provide an opportunity to address this *rehabilitation gap* and improve access to evidence-based rehabilitation alongside physical therapy at all stages of care.

**Objective:**

This study aims to understand the opinions of patients who have undergone ACL surgery and rehabilitation on the use of telerehabilitation as part of ACL care and define the population and explore their experiences and views on the acceptability of telerehabilitation after ACL reconstruction.

**Methods:**

This study was a cross-sectional, voluntary, web-based survey combining both closed and open questions. Ethical approval was obtained from the Yale School of Medicine Institutional Review Board. Participants were aged 16 years or older at the time of recruitment and had undergone ACL reconstruction within the past 5 years. A 26-item survey was developed using the Qualtrics survey platform. No items were mandatory. Responses were multiple choice, binary, and qualitative. The CHERRIES (Checklist for Reporting Results of Internet E-Surveys) was used to ensure the quality of reporting of surveys in the medical literature. Data were analyzed using Stata version 15. Qualitative data were analyzed using NVivo 11. The theoretical framework for this analysis is based on the Capability, Opportunity, and Motivation-Behavior model of behavior change.

**Results:**

A total of 100 participants opened the survey. All completers were unique. The participation and completion rates were each 96% (96/100). Patients reported their physical therapy care ended at an average of 6.4 months and that they felt fully recovered at an average of 13.2 months. Only 26% (25/96) of patients felt fully recovered at the end of physical therapy. Of these 96 patients, 54 (60%) were younger than 30 years, 71 (74%) were recreational athletes, 24 (24%) were competitive athletes, 72 (75%) had private insurance, 74 (77%) were not familiar at all with telerehabilitation, and 89% (85/96) felt capable. They preferred to use telerehabilitation at different stages of care. Reported benefits included resource saving, improved access to care, improved learning, and greater engagement. Concerns included incorrect performance of exercises or unmanaged pain being missed and less access to manual therapy, motivation, and opportunities to ask questions. Participants’ priorities for a future telerehabilitation intervention included its use as an adjunct to physical therapy rather than a replacement, with content available for each stage of care, especially return to sports. Participants stressed that the intervention should be personalized to them and include measures of progress.

**Conclusions:**

These findings helped understand and define the ACL reconstruction population. Participants found telerehabilitation acceptable in principle and highlighted the key user requirements and scope of future interventions.

## Introduction

An estimated 150,000 to 250,000 anterior cruciate ligament (ACL) injuries occur each year in the United States [1-3]. The cost is estimated at more than 3 billion dollars annually, not including the psychosocial costs incurred by patients, their families, and clinical teams involved [4]. The postoperative rehabilitation process can be lengthy [5] and includes predominantly evidence-based exercise interventions led by physical therapists through clearly defined stages of care [6-11]. It takes most patients 9 to 12 months after surgery to pass the return-to-sports criteria [5].

The return-to-sport stage of rehabilitation is crucial for those who intend to participate in sports after anterior cruciate ligament reconstruction (ACLR), particularly as returning to sports too soon risks re-injury [12,13]. It includes complex decision making around when and how to progress through physical and psychological challenges to facilitate optimum function and minimize the risk of re-injury [12,14]. It requires appropriate return-to-sport tests that recreate the physical challenges of sports in a controlled way and measure the individual’s ability to perform the necessary physical tasks of their sport [9,12,13]. Delaying return to sports until an individual passes these return-to-sport milestone tests helps prevent re-injury [3,15,16]. Negotiating this without a supervising physical therapist presents challenges, particularly as evidence suggests there is a difference between athletes’ perceived and actual readiness to return to sports [17].

A recent web-based survey of more than 1000 American physical therapists found that rehabilitation practice varied significantly in terms of the content and duration of supervised care [14]. Specifically, with regard to the length of treatment, supervised physical therapy ranged from 1 to 3 months (15% of respondents) to 12 months (11% of respondents). Overall, 56% of respondents reported 3 to 5 months or less of supervised physical therapy. These data suggest that many patients leave supervised care before the return to sports rehabilitation or achieving a return to sports [5]. This suggests the existence of a *rehabilitation gap* between the end of care and return to sports. Greenberg et al [14] concluded that this gap may contribute to patient confusion and suboptimal outcomes.

Telerehabilitation interventions may provide an option to address this rehabilitation gap. Telerehabilitation can provide evidence-based information, education, and exercise guidance and is acceptable to patients [18,19]. This technology has the potential to improve adherence to rehabilitation by engaging with mechanisms of behavior, including personalized features such as prompts, goal setting, and exercise logs [18,20]. Some of these interventions are wearable [21], some are app or website based [18,22], and some are delivered to home [19,20]. The latter study used a three-dimensional camera to facilitate communication between the physical therapists and the patients [20]. In this context, *telerehabilitation* has been employed as a catch-all term for digital rehabilitation methods and is variously called digital health, eHealth, or mobile health; the term is increasingly used to describe digital tools, including video consultation. However, in this paper, we use this catch-all term to describe digital rehabilitation strategies that may or may not include video consultations.

The rehabilitation gap appears to be caused by patients only being allowed (or able to afford) a certain number of appointments. Using telerehabilitation through the rehabilitation process as an adjunct to face-to-face care may create an opportunity to prolong physical therapy by stretching the allocated appointments over a longer period. Patients have previously used telerehabilitation for knee injuries with high fidelity and noted improved confidence and motivation with their rehabilitation [18-20]. The attitude of patients toward this technology after ACLR is unknown.

### Objectives

This study aims to understand the acceptability of patients who have completed ACL surgery and rehabilitation regarding the use of telerehabilitation as part of ACL care. Our objectives are as follows:

1. obtain patients’ experiences and opinions on their access to physical therapy throughout ACL rehabilitation,
2. explore the patients’ understanding of telerehabilitation, and
3. explore patients’ opinions about the acceptability of telerehabilitation as part of ACL care.

## Methods

### Design

We used a cross-sectional, voluntary, web-based survey combining both closed (fixed-response options) and open questions.

### Ethics

Ethical approval was obtained from the Yale School of Medicine Institutional Review Board (IRB), and proper consent procedures were employed for all participants.

### Participants and Recruitment

Potential participants were drawn from the Yale New Haven Hospital Healthcare database. Patients aged 16 years or older at the time of recruitment who had undergone ACL reconstruction within the past 5 years were invited to give their “thoughts and opinions regarding a potentially new approach to post-surgical rehab” and introduced to the possibility of telerehabilitation in addition to usual care (Multimedia Appendix 1). Potential participants were provided with an IRB-approved patient information sheet and informed that completing the survey was entirely voluntary. Consent was implied by participation. Participants were offered the opportunity to enter a draw for a US $50 Visa gift card as an expression of gratitude for their participation [23]. This was an exploratory study; therefore, no formal sample size calculation was used [23].

### Survey Development

This survey was developed using the Qualtrics survey platform. The study authors designed the questionnaire based on expertise and previous research in the field. Input on the content was sought from research and clinical peers. A total of 4 orthopedic surgeons and 5 physical therapists reviewed the questionnaire over email. This led to the removal of marginally relevant questions and the addition of questions to identify patient self-described activity levels. The questionnaire layout was organized under the guidance of a Qualtrics survey expert at Yale University. The survey was then piloted with 8 colleagues (4 physiotherapists and 4 orthopedic surgeons) and patients who had undergone ACL but were not in the study for comprehension, interpretation, and availability of appropriate responses. Questions were further edited or removed for repetition or clarification, and new response options were added. A short video example of telerehabilitation was provided.

The survey included 26 question items on the survey distributed over 4 screen pages. A total of 4 questions focused on demographic details such as age, gender, race, and socioeconomic status. The remaining questions explored participants’ experience of ACL postoperative care and their attitudes toward telerehabilitation and obtaining data on knee health. The final 3 questions of the survey invited respondents to put forward opinions about what they saw as the potential benefits and concerns of telerehabilitation and how they felt it would be best utilized in the management of patients after ACLR.

We used Likert scale answers in 2 questions. The scale chosen was a 5-point scale; however, only points 1, 3, and 5 were labeled. Points 2 and 4 were unlabeled but offered a second scale point between 1 and 3 or 3 and 5 to indicate either a slightly negative or slightly positive score that was not wholly positive or wholly negative and not neutral at 3. The challenge of measuring Likert scales is much discussed; as an ordinal scale, responses can be rated or ranked, but the space between *often* and *sometimes* cannot be empirically measured [24]. Although arguments have been made regarding parametric testing of Likert scales with normal distributions, in this instance, the Likert results are described in their ordinal categories [25].

Participants were prompted to complete questions before clicking through; no items were mandatory, which allowed participants to choose not to answer certain questions. Responses were multiple choice, binary, and qualitative. Neither randomization nor adaptive questions were used. Participants could use a *back* function to change a response if they chose to do so. A copy of the survey is available in Multimedia Appendix 2.

### Survey Distribution

The survey was distributed to patients through the Yale Qualtrics software. It was distributed on October 7, 2019, and 2 follow-up emails were sent to the nonresponders 1 week apart. Participants were advised as to the purpose of the survey.

### Data Analysis

Responses were collected through a web database. The survey responses were entered manually into a database, with the exception of time taken on the survey and percentage completed, which were captured automatically. The Checklist for Reporting Results of Internet E-Surveys, known as the CHERRIES statement, was used to ensure the quality of reporting of surveys in the medical literature [26]. The checklist is included in Multimedia Appendix 3. Eysenbach et al [26] suggested that as there is no standard methodology, authors should avoid the term *response rate*. They defined the participation and completion rates as recommended measures and calculated the participation rate as a ratio of those who click on the survey link and then chose to partake: “Count the unique number of people who filled in the first survey page (or agreed to participate, for example by checking a checkbox) divided by visitors who visit the first page of the survey.”

Data were analyzed using Stata version 15 (StataCorp). Descriptive statistics were used in the primary analysis of data to summarize the frequency and distribution of responses. Secondary analysis to determine relationships between patient demographic characteristics and multiple-choice opinion responses were performed using a chi-square test. Likert responses were analyzed in relation to patient demographic characteristics using a one-way analysis of variance (ANOVA; parametric distribution) or Kruskal-Wallis ANOVA (nonparametric distribution). Missing data were accounted for by multiple imputation techniques using means.

Qualitative questionnaire data were analyzed using NVivo 11 (QSR International). A pragmatic thematic analysis was conducted to analyze responses with respect to the research question. The process was deduced from the data driving the analysis. The themes that arose from each question were grouped so that data relating to a patient’s opinion were clear. Quotes from the survey support themes. Data are weighted and reported secondary to the frequency of occurrence or explanatory value [27]. The data were coded and analyzed independently by 2 members of the research team. Textual answers were coded into a word frequency cloud diagram that illustrates a hierarchy of terms at the heart of the participant’s responses.

The theoretical framework for this analysis is based on a model of behavior change that relates the capability, opportunity, and motivation to behavior (COM-B). [28]. In rehabilitation, the target behavior is most often exercised. Michie et al [29] designed a system in which capability, opportunity, and motivation are understood in relation to the target behavior. This model identifies that to perform the target behavior (ie, exercise), patients must be physically and psychologically capable, must have social and physical opportunities, and must be motivated to engage in behavior in the form of deeper desires and reflective planning [30]. Applying the COM-B theory to the survey results can aid further understanding of patient acceptability of telerehabilitation as a tool for ACL rehabilitation.

### Data Protection

Data were anonymized, and no personal medical data were stored or analyzed. Qualtrics survey software and the local Yale University servers were used to protect the data. Anonymized data were stored on encrypted laptops.

## Results

First, the survey response and characteristics of respondents are described, followed by the results for each objective of the study; then, the respondents’ experiences of physical therapy after ACLR, their previous experience of telerehabilitation, and views on the acceptability of telerehabilitation are reported.

### Survey Response

A total of 213 patients were contacted; 29 emails bounced and 184 were delivered. Of these, 100 respondents clicked on the survey. On the basis of internet protocol addresses, all completers were unique throughout the study. Cookies were not used to assign identifiers to each computer.

On examination of the data, 4 responses were found to be empty and were therefore excluded. Therefore, the analysis included 96 responses. The participation and completion rates were calculated according to the CHERRIES guidance [26]. For this study, the participation rate was 96% (96/100). The completion rate is calculated as the ratio of users who finished the survey relative to those who clicked on the survey link and agreed to participate. The completion rate was 96% (96/100). The view rate was not applicable. Participant characteristics are detailed in Table 1. Missing data were minimal (26/2112, 1.2%) and therefore accounted for by a multiple imputation technique using means.

**Table 1:** Demographics and patient characteristics (N=96).

Characteristics			Values
**Frequency of age at surgery (years), n (%)**			
	15-19	4 (4)	
	20-24	18 (19)	
	25-29	32 (33)	
	30-34	8 (8)	
	35-39	4 (4)	
	40-44	9 (10)	
	45-49	13 (14)	
	**>**50	8 (8)	
**Year of surgery; frequency, n (%)**			
	2014	5 (5)	
	2015	14 (15)	
	2016	27 (28)	
	2017	16 (17)	
	2018	23 (24)	
	2019	11 (11)	
**Surgical procedure; frequency, n (%)**			
	ACLR^a^ alone	57 (60)	
	ACLR with meniscus repair	36 (37)	
	ACLR with another procedure	3 (3)	
**Gender; frequency, n (%)**			
	Female	55 (57)	
	Male	40 (42)	
	Trans or nonbinary	1 (1)	
**Race/ethnicity; frequency, n (%)**			
	Asian	14 (15)	
	Black or African American	5 (5)	
	Hispanic or Latino	12 (13)	
	Native Hawaiian or other Pacific Islanders	1 (1)	
	White	64 (66)	
**Insurance; frequency, n (%)**			
	Commercial (eg, Blue Cross/Blue Shield and Cigna)	72 (75)	
	State (eg, Medicaid and Medicare)	21 (22)	
	Uninsured	3 (3)	
**Level of sport; frequency, n (%)**			
	Competitive sport/activity	24 (25)	
	I am not active	1 (1)	
	Recreational sport/activity	71 (74)	
**Returned to the same level; frequency, n (%)**			
	No	39 (41)	
	Yes	57 (59)	
**Did you have sufficient physical therapy; frequency, n (%)**			
	No	22 (23)	
	Yes	74 (77)	
**Determining factors to end physical therapy^b^** **, n (%)**			
	Determined by the expense	14 (15)	
	Determined by the insurance	36 (38)	
	Determined by the physical therapist	66 (69)	
	Determined by you	49 (51)	
	I was fully recovered	25 (26)	
	Other	12 (13)	
	Too far away	7 (7)	
	Too time consuming	18 (19)	
Percentage of knee function compared with previous, mean (SD); range			82.6 (13.8); 25-100
Months you had physical therapy, mean (SD); range			6.4 (4.8); 2-30
Patient’s stated ideal length of physical therapy (months), mean (SD); range			7.25 (4.3); 2-36
When did you feel fully recovered (months)?, mean (SD); range			13.17 (8.3); 0-60

^a^ACLR: anterior cruciate ligament reconstruction.

^b^Multiple responses were permitted; % will not add up to 100.

### Characteristics of the Respondents

More than half of patients (54/96, 56%) were younger than 30 years at the time of their surgery. There were more female respondents (55/96, 57%) than male respondents and more White respondents (64/96, 66%) than other ethnic groups. The participants identified themselves mostly as recreational athletes (71/96, 74%); 25% (24/96) identified themselves as competitive athletes, and 1% (1/96) reported that they were not active. Most participants had ACL reconstruction alone (57/96, 59%), although concomitant meniscal repair was also common (26/96, 38%). Overall, 59% (57/96) of participants reported that they returned to their pre-injury performance level. When asked to compare their knee before injury on a scale of 0 to 100, the mean knee score was 82.6 (SD 13.82). Response rates were lowest in patients who underwent surgery in 2014 but were otherwise unremarkable. Most respondents had private insurance (72/96, 75%). The results are shown in Table 1.

### Experiences of Physical Therapy

Only 26% (25/96) of patients felt that they had fully recovered at the end of their physical therapy; 77% (74/96) of patients felt that they had sufficient physical therapy, whereas 23% (22/96) did not. Moreover, 69% (66/96) of patients stated that their physical therapy was ended by their physical therapist, whereas 51% (49/96) ended physical therapy themselves, and 39% (37/96) said that travel, time commitments, and other factors were causal for ending their physical therapy Patients recalled that they had a mean of 6.4 (4.8, 2-30) months of physical therapy after ACLR. The mean preferred length of physical therapy was 7.25 (4.3, 2-36) months. The mean time to fully recover was 13.17 months. These results are shown in Table 1.

### Experience and Views on the Acceptability of Telerehabilitation: Quantitative Data

As indicated in Table 2, 92% (88/96) of patients had never used a telerehabilitation tool. Approximately 77% (74/96) reported that they were not familiar at all with telerehabilitation; 25% (24/96) of people felt there would be challenges to using telerehabilitation, although most were not overly concerned about data protection (48/96, 50% *not at all*; and 29/96, 30% were less than *somewhat concerned*). When asked in which phase they preferred to use their allocated physical therapy appointments, 60% (58/96) said they preferred face to face in the early stages of care, 33% (32/96) said they preferred to use face to face with the return-to-sport care, and 6% (6/96) chose other. Most patients (85/96, 89%) felt capable of using physical therapy, further, a secondary analysis using a chi-square test showed no significant association between capability and gender, age, level of sport, or race and ethnicity. However, we interpret this with caution, given that there was no formal sample size for this exploratory study, and there is some risk of a type 2 error.

**Table 2:** Experiences and opinions on telerehabilitation quantitative data (N=96).

Characteristics			Values
**Previous use of telerehabilitation; frequency, n (%)**			
	No	88 (92)	
	Yes	8 (8)	
**How familiar are you with telerehabilitation; frequency, n (%)**			
	1 (not familiar at all)	74 (77)	
	2	11 (12)	
	3 (somewhat familiar)	9 (9)	
	4	1 (1)	
	5 (very familiar)	1 (1)	
**Concern about data protection; frequency, n (%)**			
	1 (not at all concerned)	48 (50)	
	2	29 (30)	
	3 (somewhat concerned)	13 (14)	
	4	3 (3)	
	5 (very concerned)	3 (3)	
**How would you prioritize use of physical therapy appointments; frequency n (%)**			
	Other	6 (6)	
	Early phase	58 (61)	
	Return-to-sport phase	32 (33)	
**Do you feel capable of using telerehabilitation; frequency, n (%)**			
	No	11 (12)	
	Yes	85 (88)	
**Association of feeling capable by chi-square, *P* value**			
	Age	.94	
	Insurance	.75	
	Gender	.91	
	Race and ethnicity	.30	
	Level of sport	.79	

### Experiences and Views on the Acceptability of Telerehabilitation: Mixed Qualitative and Quantitative Data

About 25% of patients perceived challenges in using telerehabilitation at home. When participants described these challenges, 2 themes emerged and are shown in Table 3 “Resources” and “Value placed on face-to-face care.” Participant quotes are identified with the letter P and an anonymous identification number. First, access to technology such as appropriate computers with high-quality cameras for two-way communication and access to the internet were cited as potential limitations. Space and proper equipment with which to exercise were also mentioned as limitations to exercising at home. Second, patients emphasized the value they place on in-person therapy. In particular, meeting with their physical therapist afforded them the opportunity to make sure that exercise techniques were correct and that pain levels were normal. In addition, patients reported being motivated by their physical therapy and having improved confidence based on face-to-face physical therapy. They also mentioned the individualized nature of face-to-face care and the potential need for manual therapy.

**Table 3:** Experiences and opinions on telerehabilitation: mixed data.

Questions		Responses
**Are there challenges to telerehabilitation use? Frequency, n (%)**		
	No	72 (75)
	Yes	24 (25)
**If yes, what are they?**		
	Concerns about resources	“No computer.” (P10) “Lack space.” (P86) “Lack of equipment or space for exercise time management.” (P24) “not having access to equipment” (P39) “unreliable wifi” (P43)
	Value placed on face-to-face physical therapy	“Direct one on one instruction is irreplaceable.” (P11) “being able to talk through new movements” (P42) “Knowing what level of pain is ok.” (P16) “How do you know you are doing the movements correctly?” (P20) “Some of my rehab had to have been done by a physical therapist. I could not do the manipulation.” (P7)

### Experiences and Views on the Acceptability of Telerehabilitation: Qualitative Data

Within each qualitative question, patients were invited to give their opinions on telerehabilitation. Table 4 shows the 3 questions asked and the themes that arose.

Responses regarding the potential benefits of telerehabilitation were collated into 4 themes: telerehabilitation as a resource-saving companion to physical therapy care, improving access to care, a learning tool during and after physical therapy, and to engage patients more with the education and exercises of their care (Textbox 1). Patients emphasized the potential value of telerehabilitation as an adjunct to usual physical therapy care. They emphasized its potential to facilitate *saving* appointments or to fill the gap at the end of rehabilitation. Patients placed significant emphasis on how telerehabilitation could improve their access to care. They commonly cited a lack of transport options, cost of transport, or bad weather as limiting their access, and they saw telerehabilitation as a way to improve this. Patients who had concluded physical therapy believed that it has a potential benefit of having access to telerehabilitation, such as an app, in an ongoing way to provide maintenance advice and reminders. Respondents identified their understanding of the role of telerehabilitation in motivating and engaging them in personal care plans. Goal setting, progress measures, and modes of encouragement were all mentioned as benefits of using telerehabilitation.

**Table 4:** Experience and opinions of telerehabilitation: qualitative data.

Question	Theme 1	Theme 2	Theme 3	Theme 4
What, if any, do you see as potential benefits to the use of telerehabilitation following ACL^a^ reconstruction surgery?	Companion to physical therapy	Improving access to care	Telerehabilitation as a learning tool over the long term	Telerehabilitation as a tool to motivate and engage
What, if any, are your primary concerns regarding the use of telerehabilitation following your ACL reconstruction surgery?	Clinical concerns: biopsychosocial needs of patient	Sociopolitical	—^b^	—
What would you need from a telerehabilitation program to make it right for you to use?	Utilized alongside physical therapy	Preferred timing of telerehabilitation use	Personalized	—

^a^ACL: anterior cruciate ligament.

^b^Themes were not present for all questions.

**What are the potential benefits of telerehabilitation? (PT: physical therapy).**

Question asked: What, if any, do you see as potential benefits to the use of telerehabilitation following anterior cruciate ligament reconstruction surgery?

- Telerehabilitation as a resource-saving companion to physical therapy care:
  “It might be useful for the home exercises, but would have to be paired simultaneously with in-person treatment to be useful.” (P24)
  “Also you could save your Dr visits when it counts the most.” (P10)
  “Physical therapy was mostly for the initial recovery (for the first three months at most). I wish I had more sessions for ‘return-to-sport’ type exercises. Of course being supervised would be ideal but perhaps ‘return-to-sport’ type rehab can be done remotely.” (P19)
  “For patients who have a time constraint and who have run out of in person rehab, this may be a more affordable option.” (P20)
  “Saving rehabilitation appointments until later.” (P60)

- Telerehabilitation as a resource to improve access to care:
  “Tele-rehab would save the commute from home or class to a PT center, which is difficult directly post-surgery, and especially so if the patient (such as a student) lives on their own.” (P83)
  “In case you are not able to make it to your appointments.” (P2)
  “Reduction in commuting costs.” (P68)

- Telerehabilitation as a learning tool during and after physical therapy:
  “Don’t know what the correct exercises are now to keep strengthening it. I would love to have specific exercises so I would go on your tele rehab app now.” (P23)
  “Improvement in return to activity and return to full prior level of activity—and maintenance after.” (P21)
  “I felt like I was released from PT before my recovery was ‘complete,’ but I had passed the tests. This would hopefully help prevent that from happening.” (P13)

- Telerehabilitation to motivate and engage patients:
  “I would use tele-rehab daily, because having a program that measures your progress and fulfilment of daily goals is more encouraging than a doctor’s note that tells you to do ‘x’ repetitions of some exercise, which I would be less likely to do regularly.” (P83)

Patient concerns with telerehabilitation predominantly focused on the clinical issues of patients and their biopsychosocial needs (Textbox 2). They were concerned about a negative impact on their care if they were performing exercises incorrectly or if they were not managing their pain well. Access to manual therapy, motivation, and opportunities to ask questions were again cited as reasons why they would require one-on-one sessions in addition to telerehabilitation. One patient expressed a strong opinion that telerehabilitation was providing a solution to the problem of not enough health care insurance. One participant described wanting to be able to progress at her own rate. Others commented simply that it should be personalized or specific.

**What are your potential concerns about the use of telerehabilitation in care?**

Question asked: What, if any, are your primary concerns regarding the use of telerehabilitation following your anterior cruciate ligament reconstruction surgery?

- Clinical concerns: biopsychosocial needs of patient
  “In the initial recovery phase, I wouldn’t be confident enough to judge what’s OK and what’s not.” (P19)
  “It would slow rehab process due to lack of personal contact.” (P11)
  “No instructor there to tell you if you are doing the movements incorrectly. I know there is a camera but does it pick up on minor nuances?” (P20)
  “Not pushing myself especially if there is pain.” (P84)
  “I need someone to push me.” (P88)

- Sociopolitical concerns
  “The state of healthcare in this country is deplorable. If the reason for a tele-rehab program is that insurance won’t cover enough in person visits, the insurance system needs to be fixed, not the treatment system.” (P24)

The final question was, “What would you need from a telerehabilitation program to make it right for you to use?” The predominant theme was that telerehabilitation needed to be used as an adjunct to physical therapy rather than instead of it. Further themes focused on the *preferred timing* of a telerehabilitation tool and a necessity to be personalized so that a patient’s individual plan is reflected in their care needs (Textbox 3).

Patients discussed the necessity of progress testing and supervision of physical therapy as important to their recovery and outcomes. They re-emphasized the value they place on face-to-face care and had differing views on whether they would prefer to use telerehabilitation in different stages of care. Many patients expressed that they would prefer to have access to face-to-face rehabilitation in the early stages of care. Some patients emphasized the appeal of having return-to-sports content on a telerehabilitation tool and discussed telerehabilitation as being adaptive to their individual needs.

**What would you need from a telerehabilitation program to make it right for you to use? (PT: physical therapy).**

Question asked: What would you need from a telerehabilitation program to make it right for you to use?

- Utilized alongside physical therapy
  “I would need to intersperse the program with visits to an actual PT centre, where therapists would check in on my progress and either tell me to go harder or back off on the intensity of the exercises.” (P83)
  “I wouldn’t have it replace all face to face appointments, but I would think it might be a good idea to use on every other appointment.” (P68)

- Preferred timing of telerehabilitation use
  “Mid-late phase is ok.” (P11)
  “If it can help me with the ‘return-to-sport’ type exercises (at various levels), I would be interested.” (P19)
  “I would like to use it after the first month or two of in person rehab.” (P20)
  “I would prefer to have (a) professional at least in early stages of recovery process rather than tele-rehabilitation.” (P63)

- Personalized
  “I would want to have the option to make exercises more difficult: younger bodies recover faster than older ones, and I was often frustrated during my therapy by how basic some exercises were, especially early during the recovery.” (P83)
  “Specific*.*” (P9)

## Discussion

### Principal Findings

These results provide a detailed impression of participants’ knee health, their recollections of access to physical therapy, and their opinions on the role of telerehabilitation in ACL rehabilitation. Missing data were minimal, and the results demonstrated that for many patients, a rehabilitation gap exists between the time they are discharged from care and the time they recovered. It is unlikely that an average patient would have passed the return-to-sport criteria when care was completed at 6 months, given that 9 and 12 months have been given as typical [5,12,31]; return to sports before 9 months increases the risk of re-injury by 51% [13], and returning to sports without meeting the specific physical criteria also increases the risk of re-injury [16]. It is, therefore, highly likely that patients, including those in this cohort, are discharged from care before they can undertake advanced rehabilitation or be subjected to the return-to-sport criteria testing, and this may expose them to an increased risk of re-injury.

Recovery after ACLR was also measured by a return to the previous level of activity. Feucht et al [32] reported that 91% of athletic patients expect to return to the same level of sport. In this study, 99% of patients identified as being either competitively or recreationally athletic, indicating that the goal of return to sports was likely commonplace. Typically, 55% of patients returned to compete in sports at the same level as before their injury [31]. In line with these findings, 59% of our study respondents reported return at the same level. Although the overall reported knee health was 83% of the pre-injury level, this figure is difficult to interpret because of its subjectivity and demonstrates the importance of objective outcome measures such as the Knee Injury and Osteoarthritis Outcome Score (KOOS), which can indicate in which area of function or daily life the knee deficit persists [33]. The data in this study contributed to the knowledge that patients undergoing ACLR see themselves as competitive or recreationally athletic, and a lack of guidance during the advanced rehabilitation and return-to-sports phase could contribute to overall suboptimal outcomes for return to previous levels of activity [14].

When considering participants’ access to physical therapy, there are some contradictory responses for which we can suggest possible explanations. The finding that 77% of patients felt that they had sufficient physical therapy appears to contradict the finding that only 11% of patients felt that they had fully recovered at the end of physical therapy. The possible explanations for this are that patients were satisfied with the level that they had achieved despite not reaching full recovery or that they continued to progress through rehabilitation independently or with support from outside physical therapy such as athletic trainers, coaches, or personal trainers.

It is also important to note that some patients may choose not to return to sports, and older patients (30% of respondents were older than 40 years) were more likely than younger patients to choose to modify their activities rather than returning at the same level [34]. The fear of re-injury is frequently cited as a cause of people not returning to sports [35]. Some participants with less physically challenging goals may have completed their rehabilitation at 6 months; however, this does not include people who are returning to high-demand sports such as football, lacrosse, and soccer.

The aim of this study is to investigate the acceptability of telerehabilitation tools in ACL rehabilitation. It is thought that telerehabilitation may have positive benefits for delivering exercise interventions and create opportunities for improved self-management and progress measurement [36]. There is growing availability and popularity of health apps and other telerehabilitation tools [37,38]; however, they have not been widely integrated into rehabilitation practice or tested under research conditions. As such, patient experience and knowledge may be minimal. Indeed, our data suggested that 92% had never used telerehabilitation. Patients in this study primarily expressed concern over how telerehabilitation is integrated into current care, with an emphasis on not wanting telerehabilitation to replace face-to-face care but to be offered alongside it. Concern about the security of their data was less of an issue for this cohort (20%).

The COM-B model of behavior change suggests that having the capability, motivation, and opportunity to perform a given task is associated with behavior outcomes [29]. In rehabilitation, the target behavior is often the exercise. This analysis considered participants’ survey responses in relation to their understanding of exercise and rehabilitation behavior. Participants were not concerned about being able to use telerehabilitation; 89% of the patients felt that they were capable, indicating that they would have the knowledge and skill to engage with this process. There was no indication of a digital divide among respondents, with no significant interaction being found between capability and participant demographic group (Table 3); however, digital literacy may have excluded some people from a web survey at the first instance. One-fourth (25%) of patients who expressed concern were predominantly focused on having the physical capability or skill to perform exercises correctly without the supervision of a physical therapist. Any implemented telerehabilitation tool would need to address this concern through careful development.

Opportunities to change behavior can be both physical and social. Physical opportunities for rehabilitation include the availability of resources and the environment to facilitate behavior. A social opportunity might pertain to cultural norms and standards that are familiar. About 25% of patients identified potential physical opportunity challenges to using telerehabilitation, such as access to computers, physical space, and Wi-Fi. They further referenced social norms and beliefs about the values of traditional physical therapy. They expressed anxiety about how telerehabilitation might be integrated into care and potential loss of face-to-face therapy. About 60% of patients expressed that they would prefer to use fewer appointments in the early stage of their care and *save* them for later, whereas the remaining identified that they would prefer to use telerehabilitation in the return-to-sports phase.

Patients also endorsed opportunities to adapt to norms. They highlighted the potential benefits of telerehabilitation alongside physical therapy to improve care and create physical opportunities such as saving time and money by avoiding commutes. A number of patients also identified the potential of telerehabilitation as a maintenance tool after discharge from physical therapy, whereas some reported that they felt their care ended prematurely.

Motivation to exercise is a significant factor in rehabilitation behavior, and, similar to other psychological factors, it is positively correlated with successful outcomes in ACL rehabilitation [39,40]. Digital health tools are thought to influence motivation and adherence to exercise programs [18,41,42]. Motivation can be reflective, including goals and plans, and automatic, where it interacts with wants, needs, and impulses [29]. One participant stated that being able to set and fulfill their own goals through telerehabilitation would be “more encouraging.” They discussed the benefit of measures of progress, which is a common function of telerehabilitation tools, where they can collect outcomes such as KOOS and pain scores to measure and record progress over time and motivate the patient to continue to set and pursue goals in a validated way [18,20].

### Limitations

Limitations of this survey include the potential for recollection bias with regard to how much physical therapy patients had, and participants who had undergone surgery more recently may have been less able to offer a long-term view. The study had a relatively small sample size, and, as an exploratory study, psychometric properties were not calculated, which may inform the reliability of results.

All surveys are limited to the data gathered, and it is known that participation can be affected by socioeconomic condition and education or digital literacy levels, where some populations are known to be less likely to access internet surveys [43]. This may include a population for whom telerehabilitation is not appropriate, and therefore, face-to-face rehabilitation would need to be provided. Future work should include a measure of insurance type in the data set where possible so that socioeconomic biases can be identified. For example, this study had few Medicaid patient responses, but it is not known whether this is related to fewer invites issued to Medicaid patients or if it could be considered to be related to socioeconomic disadvantage where Medicaid patients are known to be at a greater risk [44-46].

### Conclusions

Physical therapy care most commonly ends before patients reach the return-to-sport phase of care. This indicates that there is a rehabilitation gap where patients may not have access to the best guidance for return-to-sports rehabilitation. This may have implications for their injury risk and successful return to sports. Telerehabilitation may provide an alternative way for patients to access evidence-based ACL rehabilitation in this phase. The results suggest that telerehabilitation is acceptable to patients as part of their rehabilitation following ACLR but would need to be an adjunct to care rather than a replacement.

## Appendix

Multimedia Appendix 1 Introductory email.

Multimedia Appendix 2 Survey.

Multimedia Appendix 3 CHERRIES (Checklist for Reporting Results of Internet E-Surveys).

## Abbreviations

ACL: anterior cruciate ligament
ACLR: anterior cruciate ligament reconstruction
ANOVA: analysis of variance
CHERRIES: Checklist for Reporting Results of Internet E-Surveys
COM-B: Capability, Opportunity, and Motivation-Behavior
IRB: institutional review board
KOOS: Knee Injury and Osteoarthritis Outcome Score
NIHR: National Institute for Health Research
