# Supplementary material for: Telerehabilitation to Address the Rehabilitation Gap in Anterior Cruciate Ligament Care: Survey of Patients
Source: JMIR Form Res. 2020 Sep 18;4(9):e19296. doi: 10.2196/19296 (PMC7532455; doi:10.2196/19296)
Supplement: Multimedia Appendix 1 [file formative_v4i9e19296_app1.docx]

Multimedia Appendix 1

Dear ${m://FirstName},

I hope that you are doing well.  I am writing to invite you to participate in an important survey I am conducting regarding rehabilitation after ACL surgery like yours.  You may have been my patient or a patient of my colleague Dr ******. Through this study I hope to understand your opinions and thoughts regarding a potentially new approach to post-surgical rehab, which I hope could improve the experience and outcome for patients like you.

The survey should take approximately 15 minutes to complete.

In exchange for your time and participation, you will have the opportunity to be entered into a draw to win one of 2 $50 Visa Gift Cards.

If you have any questions about this survey, please feel free to contact research associate/physical therapist Emma Dunphy at emma.dunphy@yale.edu.

Thank you for your time and input, and hope that you and your knee continue to do well!

Elizabeth Gardner, MD

Yale University Department of Orthopaedic Surgery

Sports Medicine Surgeon

**Follow this link to the Survey:**
${l://SurveyLink?d=Take the Survey}

Or copy and paste the URL below into your internet browser:
${l://SurveyURL}

Follow the link to opt out of future emails:
${l://OptOutLink?d=Click here to unsubscribe}
