# Supplementary material for: Telerehabilitation to Address the Rehabilitation Gap in Anterior Cruciate Ligament Care: Survey of Patients
Source: JMIR Form Res. 2020 Sep 18;4(9):e19296. doi: 10.2196/19296 (PMC7532455; doi:10.2196/19296)
Supplement: Multimedia Appendix 2 [file formative_v4i9e19296_app2.docx]

Multimedia Appendix 2

ACL Retrospective Patient Survey

Start of Block: General Information

Q33 Thank you for participating in the ACL survey. For information about the survey, please click here: [Patient Information Sheet](https://yalesurvey.ca1.qualtrics.com/CP/File.php?F=F_6s1POrkyrASptDn)

Ret_age_surgery What age were you at the time of your surgery?

- under 15 (9)
- 15-19 (1)
- 20-24 (2)
- 25-29 (3)
- 30-34 (4)
- 35-39 (5)
- 40-44 (6)
- 45-49 (7)
- over 50 (8)

Ret_year_surgery What year was your surgery?

- 2013 (1)
- 2014 (2)
- 2015 (3)
- 2016 (4)
- 2017 (5)
- 2018 (6)
- 2019 (7)

Ret_other_surgery Which best describes your ACL surgery?

- ACL reconstruction alone (1)
- ACL reconstruction with meniscus repair/debridement (2)
- ACL reconstruction with another procedure (3)

Ret_gender What is your gender?

- Female (1)
- Male (2)
- Trans/Non-binary (4)

Ret_race_ethnicity What is your race/ethnicity?

- American Indian or Alaska Native (1)
- Asian (2)
- Black or African American (3)
- Hispanic or Latino (4)
- Native Hawaiian or Other Pacific Islander (5)
- White (6)

Ret_insurance Who was your insurance provider at the time of surgery?

- Commercial (i.e. BCBS, Cigna) (1)
- State (Medicaid, Medicare) (2)
- Uninsured (3)

| 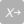 |
| --- |

Ret_level_of_sport What level of sport did you play before your injury?

- Competetive Sport/Activity (0)
- Recreational Sport/Activity (2)
- I am not active (3)

| 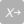 |
| --- |

Ret_return_same Did you return to the same level of sport/activity after your ACL reconstruction?

- Yes (1)
- No (0)
- N/A (2)

Ret_knee_compare Compared to your knee prior to your ACL injury, what is the function of your knee today (with 100% being the same or  better, and 0% being non-functional)?  Please use the slide to indicate current function.

|  | 0 | 10 | 20 | 30 | 40 | 50 | 60 | 70 | 80 | 90 | 100 |
| --- | --- | --- | --- | --- | --- | --- | --- | --- | --- | --- | --- |

| Percent Functional () | 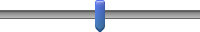 |
| --- | --- |

Ret_how_long_pt How long did your physical therapy last after your ACL reconstruction surgery?

- Number of months (4) ________________________________________________

Ret_how_long_recover When did you feel that you had fully "recovered" from your ACL reconstruction surgery?

- Number of months (4) ________________________________________________

| 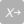 |
| --- |

Ret_sufficient_pt Did you feel that you had sufficient supervised physical therapy after your ACL reconstruction surgery?

- Yes (1)
- No (0)

Ret_ideal_length How long do you think that physical therapy should last after ACL reconstruction surgery for the average patient?

- Number of months (4) ________________________________________________

Ret_determin_factors What do you think will cause your physical therapy to end? Choose as many as you think are relevant to you.

- Determined by the physical therapist (1)
- Determined by you (2)
- Determined by the insurance (3)
- Determined by the expense (4)
- Too time consuming (5)
- Too far away (6)
- I was fully recovered (7)
- Other (8) ________________________________________________

| Page Break |  |
| --- | --- |

End of Block: General Information

Start of Block: Attitudes

| 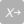 |
| --- |

Ret_prev_use_tr Have you or anyone you know used tele-rehabilitation such as apps,  websites or computer software to help with rehabilitation after any surgery or injury?  If yes, please explain

- Yes (1)
- No (0)

Ret_familiar_tr How familiar are you with tele-rehabilitation programs for any injury or condition?

- 1 - Not Familiar At All (1)
- 2 (2)
- 3 - Somewhat Familiar (3)
- 4 (4)
- 5 - Very Familiar (5)

Q32 **The following clip is given as an example of tele-rehabilitation. The programme is called VERA and is a product of Reflexion Health. View the video to see how it teaches exercise and uses a 3D camera to give the patient feedback. It has been shown to be effective and popular with patients following hip and knee surgery.**

Q11

| Page Break |  |
| --- | --- |

| 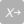 |
| --- |

Ret_capable_tr Do you feel capable of using a tele-rehabilitation program?

- Yes (1)
- No (0)

Ret_challenges_home Are there any potential challenges for you to have used tele-rehabilitation at home?  Please explain.

- Yes (1) ________________________________________________
- No (2) ________________________________________________

Ret_4weeks Would you be willing to utilize tele-rehabilitation for the first 4 weeks of your rehabilitation after ACL reconstruction surgery? You would have a device set up in your home and direct contact with a physical therapist.

- 1 - Not Willing (1)
- 2 (2)
- 3 - Somewhat Willing (3)
- 4 (4)
- 5 - Definitely Willing (5)

Ret_sensitive_info How concerned are you about the potential leak of sensitive medical information during your use of tele-rehabilitation?

- 1 - Not at all Concerned (1)
- 2 (2)
- 3 - Somewhat Concerned (3)
- 4 (4)
- 5 - Very Concerned (5)

Ret_early_goals_tr As you may remember, the primary goals of the first 4 weeks after ACL reconstruction surgery are to decrease your swelling, regain movement of your knee and get your muscles working again with gentle exercises so they will support your leg when you walk.  With this in mind, would you be comfortable to achieve these goals at home via tele-rehabilitation?

- 1 - Not At All (1)
- 2 (2)
- 3 - Somewhat Possible (3)
- 4 (4)
- 5 - Definitely Possible (5)

Ret_import_face2face How important do you view the face to face contact with a physical therapist to be in the first 4 weeks of your rehabilitation after ACL reconstruction surgery?

- 1 - Not Very Important (1)
- 2 (2)
- 3 - Somewhat Important (3)
- 4 (4)
- 5 - Very Important (5)

Ret_prioritise Unfortunately, patients often run out of physical therapy appointments before rehabilitation is complete. If you had to prioritise the use of face to face appointments would you prefer to use them for the early post operative phase or during the later return to sports phase?

- The Early Phase (1)
- The Return to Sports Phase (2)
- Other (3)

| Page Break |  |
| --- | --- |

Q25 **For the following 3 questions, we want to hear your thoughts on tele-rehabilitation.**

Ret_what_tr_needs What would you need from a tele-rehabilitation program to make it right for you to use?

________________________________________________________________

________________________________________________________________

________________________________________________________________

________________________________________________________________

________________________________________________________________

Ret_concerns_tr What, if any, are your primary concerns regarding the use of tele-rehabilitation following your ACL reconstruction surgery?

________________________________________________________________

________________________________________________________________

________________________________________________________________

________________________________________________________________

________________________________________________________________

Ret_benefits_tr What, if any, do you see as potential benefits to the use of tele-rehabilitation following ACL reconstruction surgery?

________________________________________________________________

________________________________________________________________

________________________________________________________________

________________________________________________________________

________________________________________________________________

Q34
In appreciation of your time, please click the link below to enter the draw for a chance to win one of five $50 Visa gift cards.  Your ACL tele-rehabilitation survey remains anonymous.
  <https://yalesurvey.ca1.qualtrics.com/jfe/form/SV_8jglHFmVTuZNQ9f>

End of Block: Attitudes
